# Pharmacophore Modeling for Anti-Chagas Drug Design Using the Fragment Molecular Orbital Method

**DOI:** 10.1371/journal.pone.0125829

**Published:** 2015-05-11

**Authors:** Ryunosuke Yoshino, Nobuaki Yasuo, Daniel Ken Inaoka, Yohsuke Hagiwara, Kazuki Ohno, Masaya Orita, Masayuki Inoue, Tomoo Shiba, Shigeharu Harada, Teruki Honma, Emmanuel Oluwadare Balogun, Josmar Rodrigues da Rocha, Carlos Alberto Montanari, Kiyoshi Kita, Masakazu Sekijima

**Affiliations:** 1 Global Scientific Information and Computing Center, Tokyo Institute of Technology, Meguro, Tokyo, 152–8550, Japan; 2 Graduate School of Agricultural and Life Sciences, The University of Tokyo, Bunkyo, Tokyo, 113–8657, Japan; 3 Department of Computer Science, Tokyo Institute of Technology, Meguro, Tokyo, 152–8550, Japan; 4 Graduate School of Medicine, The University of Tokyo, Bunkyo, Tokyo, 113–0033, Japan; 5 Chemistry Research Labs, Drug Discovery Research, Astellas Pharma Inc., Tsukuba, Ibaraki, 305–8585, Japan; 6 Graduate School of Pharmaceutical Sciences, The University of Tokyo, Bunkyo, Tokyo, 113–0033, Japan; 7 Graduate School of Science and Technology, Kyoto Institute of Technology, Sakyo, Kyoto, 606–8585, Japan; 8 Department of Biochemistry, Ahmadu Bello University, Zaria, 2222, Nigeria; 9 Instituto de Química de São Carlos, Universidade de São Paulo, São Carlos, 13566–590, Brazil; Instituto Butantan, Laboratório Especial de Toxinologia Aplicada, BRAZIL

## Abstract

**Background:**

Chagas disease, caused by the parasite *Trypanosoma cruzi*, is a neglected tropical disease that causes severe human health problems. To develop a new chemotherapeutic agent for the treatment of Chagas disease, we predicted a pharmacophore model for *T*. *cruzi* dihydroorotate dehydrogenase (TcDHODH) by fragment molecular orbital (FMO) calculation for orotate, oxonate, and 43 orotate derivatives.

**Methodology/Principal Findings:**

Intermolecular interactions in the complexes of TcDHODH with orotate, oxonate, and 43 orotate derivatives were analyzed by FMO calculation at the MP2/6-31G level. The results indicated that the orotate moiety, which is the base fragment of these compounds, interacts with the Lys43, Asn67, and Asn194 residues of TcDHODH and the cofactor flavin mononucleotide (FMN), whereas functional groups introduced at the orotate 5-position strongly interact with the Lys214 residue.

**Conclusions/Significance:**

FMO-based interaction energy analyses revealed a pharmacophore model for TcDHODH inhibitor. Hydrogen bond acceptor pharmacophores correspond to Lys43 and Lys214, hydrogen bond donor and acceptor pharmacophores correspond to Asn67 and Asn194, and the aromatic ring pharmacophore corresponds to FMN, which shows important characteristics of compounds that inhibit TcDHODH. In addition, the Lys214 residue is not conserved between TcDHODH and human DHODH. Our analysis suggests that these orotate derivatives should preferentially bind to TcDHODH, increasing their selectivity. Our results obtained by pharmacophore modeling provides insight into the structural requirements for the design of TcDHODH inhibitors and their development as new anti-Chagas drugs.

## Introduction

Chagas disease is an infectious disease caused by the parasitic protozoan *Trypanosoma cruzi* (*T*. *cruzi*) and is the third most common parasitic disease in the world [1]. It affects people from approximately 20 countries, particularly those living in the southern United States and Latin America [2–4], with 15 million people estimated to be infected [5]. *T*. *cruzi* is primarily transmitted by blood-sucking insects belonging to the subfamily *Triatominae* (family *Reduviidae*) or by infected blood transfusion [6]. Once infected, the host may experience influenza-like symptoms during the acute phase and gastrointestinal and cardiac disorders during the chronic phase [7–8]. Two drugs, nifurtimox and benznidazole, are currently available for the treatment of Chagas disease, but there are severe problems associated with their use, including adverse effects and limited effectiveness during the chronic phase of the disease [9–10]. Thus, developing new therapeutic agents against *T*. *cruzi* infection is desirable [11–12].

To develop a novel anti-Chagas drug, we focused on dihydroorotate dehydrogenase (DHODH) as the target protein. DHODH is an enzyme that takes part in the fourth step in the *de novo* biosynthesis of pyrimidines, which are heterocyclic compounds essential for RNA and DNA synthesis. This enzyme is an oxidoreductase that catalyzes the oxidation of dihydroorotate to orotate using flavin mononucleotide (FMN) as a cofactor. FMN can take either an oxidized form (FMN) or a reduced form (FMNH_2_), and the oxidized form serves as the oxidizing agent during orotate production. FMNH_2_ is re-oxidized by an electron acceptor that differs according to the cellular localization of DHODH [13]. In humans, DHODH is a mitochondrial inner-membrane protein that uses respiratory ubiquinone as the electron acceptor [14]. In contrast, *T*. *cruzi* DHODH (TcDHODH), a cytosolic protein, uses fumarate as the electron acceptor [15]. A previous study showed that a DHODH-knockout *T*. *cruzi* was not viable [16]. The differences in biochemical properties between human and *T*. *cruzi* DHODHs as well as its essentiality for the parasite make TcDHODH a promising target for developing novel therapeutic agents against Chagas disease.

DHODH is a validated drug target for humans [17–18], as an immunosuppressant and also against *Plasmodium falciparum* [17, 19, 20] and *Helicobacter pylori* [21]. Species-specific DHODH inhibitors have been developed and shown to be effective *in vitro* [22] and *in vivo* [23]. However, all inhibitors developed to date target the ubiquinone binding site and do not inhibit the cytosolic DHODHs, for which potent and selective inhibitors have never been reported.

**Fig 1:**
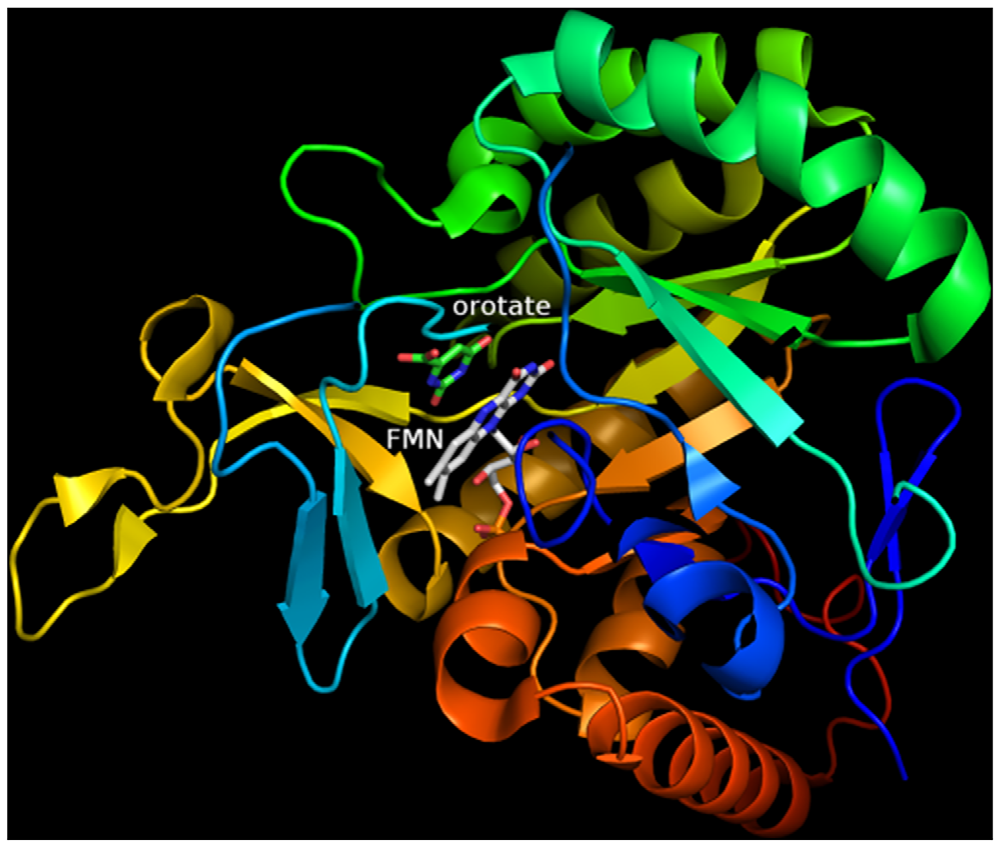
TcDHODH overall structure. Crystal structure of TcDHODH (PDB ID: 2E6A, A-chain).

The atomic resolution crystal structures of TcDHODH in complexes with its substrates and products have been determined [15]. Based on analysis of overall structure and the active site region of the TcDHODH-orotate complex (Fig 1), it is thought that a strong π-π interaction between orotate and the isoalloxazine ring of FMN occurs.

A pharmacophore is defined as “an ensemble of steric and electronic features that ensures optimal supramolecular interactions with a specific biological target and the trigger (or inhibit) of its biological function” [24]. Based on this definition, we define pharmacophore modeling as a process for predicting pharmacophores with common or specific characteristics among compounds. This definition is applied not only to molecular design but to protein—ligand docking simulation and quantitative structure-activity relationships (QSAR) as well [25]. However, pharmacophore modeling without ligand structural alignment information is difficult. Thus, knowledge of protein—ligand structure is useful for predicting pharmacophores.

The fragment molecular orbital (FMO) method [26] employs *ab initio* quantum mechanical calculations for large biomolecules such as protein—ligand complexes. Intermolecular interaction energies typically can be determined on the basis of molecular mechanics. However, this method is not universally applicable to all compounds, because there is a limit to the determination of molecular potentials based on atom type, especially of quantum chemical elements such as π electrons. For this reason, in this study, we used the FMO method to analyze the interaction energies between the target proteins and ligands with the aim of identifying important amino acid residues for ligand binding. Amino acids and ligands in the system of interest are divided into fragments, and molecular orbital calculations are performed for individual fragments. Because the effects of interfragment potentials are taken into account in these molecular orbital calculations, the FMO method can estimate the interaction energy between each pair of fragments. The method can clearly describe the detailed interactions between the ligand and each amino acid residue, and is frequently used in the design of new drugs [27–34]. Moreover, the method can extract specific interaction from a wide variety of derivatives. FMO calculation is thus suitable for obtaining pharmacophore models and is useful for guiding molecular design.

In the present study, we identified pharmacophores using the FMO method with the aim of designing anti-Chagas drugs, via analysis of interaction energy between TcDHODH and orotate, oxonate [35] as a competitive inhibitor of DHODH, and 43 orotate derivatives.

## Materials and Methods

### Co-crystallization of TcDHODH with orotate derivatives

**Fig 2:**
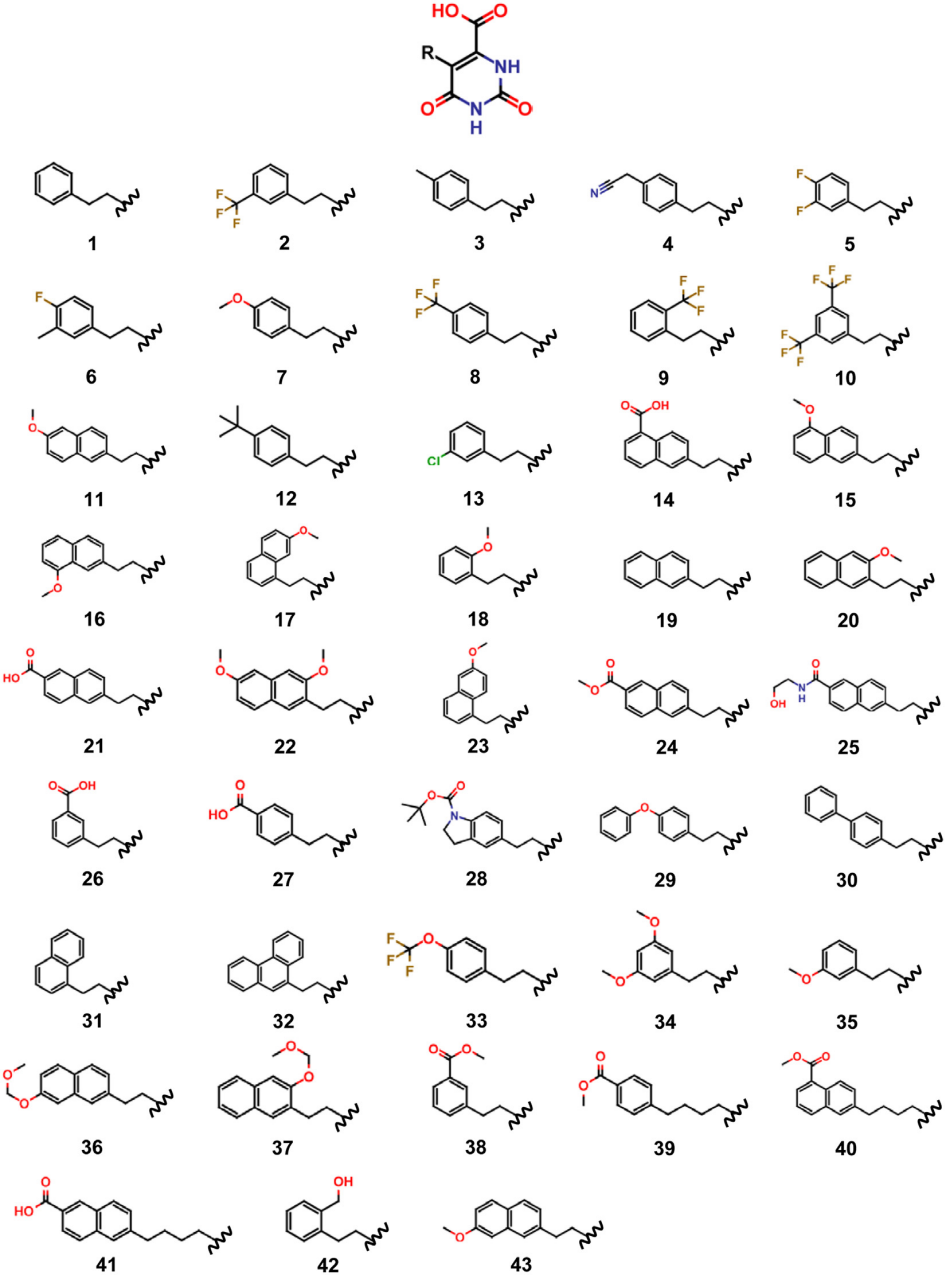
Structures of orotate and R groups of synthesized derivatives. Functional groups were introduced at the pyrimidine 5-position through an ethylene or butylene linker.

Recombinant TcDHODH expression, purification, and crystallization in complex with 43 orotate derivatives were performed essentially as previously reported [15]. Briefly, TcDHODH was purified to homogeneity from BL21(DE3)pET3a/TcDHODH using DEAE Sepharose Fast Flow (GE Healthcare) followed by Phenyl Sepharose H.P. (GE Healthcare) and TSK G3000SW (Tosoh). By this method, a total of 10–15 mg of TcDHODH with specific activity ranging from 12 to 18 μmol/min/mg could be purified from 10 L of culture. TcDHODH crystals formed in the presence of oxonate [15] were used to soak overnight into crystallization buffer containing 1 mM of orotate derivatives that are listed in Fig 2. X-ray diffraction data were collected at SPring-8 (beam lines BL32XU, BL41XU and BL44XU) or Photon Factory (beam lines AR-NW12A, AR-NE3A, BL-5A and BL-17A). All the data were processed and scaled with HKL2000 [36]. The co-crystal structures of TcDHODH in complex with orotate derivatives were initially solved by molecular replacement using coordinates from TcDHODH-oxonate complexed structure (3W1Q) and later by one of the open form structures (e.g., 3W1R). Manual building and crystallographic refinement were performed with the programs COOT [37] and REFMAC5 [38], respectively. The PDB IDs, resolution, R-value, R-Free, average of the occupancy of the ligands and average of the B-factors are listed in Table 1. The detailed synthetic process of the 43 orotate derivatives and the X-ray analysis performed in this study will be described elsewhere (Inaoka *et al*., manuscript in preparation).

**Table 1:** Summary of co-crystal structures (UniProtKB: Q4D3W2).

PDB ID	Derivative	Resolution(Å)	R-value	R-Free	Ligand Occupancy	B-factor (ligand)	B-factor (All)
3W1R	**1**	1.58	0.157	0.181	1.00	12.92	11.45
3W1T	**2**	1.68	0.144	0.179	1.00	15.02	12.69
3W1U	**3**	1.85	0.153	0.185	1.00	18.78	15.90
3W1X	**4**	1.45	0.140	0.161	1.00	8.31	11.01
3W2J	**5**	1.42	0.144	0.168	1.00	13.82	11.56
3W2K	**6**	1.54	0.138	0.169	1.00	19.46	13.26
3W2L	**7**	1.64	0.136	0.170	1.00	16.30	13.27
3W2M	**8**	1.58	0.139	0.169	1.00	15.00	12.37
3W2N	**9**	1.96	0.149	0.194	1.00	10.98	14.11
3W2U	**10**	2.25	0.208	0.270	1.00	23.15	22.87
3W3O	**11**	1.96	0.151	0.192	1.00	19.84	20.06
3W22	**12**	1.98	0.166	0.211	1.00	26.97	23.73
3W23	**13**	1.48	0.146	0.177	1.00	16.13	13.79
3W6Y	**14**	2.68	0.199	0.282	1.00	40.63	23.14
3W7C	**15**	1.75	0.144	0.195	1.00	14.68	13.99
3W7D	**16**	1.52	0.146	0.174	1.00	14.87	13.93
3W7E	**17**	1.56	0.143	0.173	1.00	13.05	12.60
3W7G	**18**	1.55	0.138	0.165	0.67	11.01	12.85
3W7H	**19**	1.67	0.145	0.176	0.67	18.94	15.19
3W7I	**20**	1.69	0.145	0.179	1.00	19.22	13.84
3W7J	**21**	1.58	0.149	0.182	0.67	10.33	10.79
3W7K	**22**	1.61	0.150	0.180	1.00	15.93	15.69
3W7L	**23**	1.88	0.145	0.189	1.00	14.73	14.66
3W7M	**24**	2.40	0.175	0.246	1.00	33.44	29.54
3W7N	**25**	2.39	0.188	0.255	1.00	53.08	37.34
3W7O	**26**	1.68	0.142	0.178	1.00	14.63	13.76
3W7P	**27**	1.70	0.142	0.175	1.00	18.19	13.29
3W7Q	**28**	1.83	0.144	0.179	1.00	19.10	18.48
3W70	**29**	2.60	0.202	0.275	1.00	42.47	25.34
3W71	**30**	1.68	0.146	0.175	1.00	21.25	15.98
3W72	**31**	1.55	0.142	0.172	0.67	11.48	14.44
3W73	**32**	1.78	0.146	0.181	1.00	19.84	14.40
3W74	**33**	1.90	0.175	0.228	1.00	29.90	22.87
3W75	**34**	1.47	0.139	0.166	0.67	12.02	12.50
3W76	**35**	1.58	0.140	0.170	1.00	16.11	14.47
3W83	**36**	2.80	0.199	0.297	1.00	40.63	23.70
3W84	**37**	1.93	0.202	0.254	1.00	22.26	16.84
3W85	**38**	2.00	0.157	0.202	1.00	24.14	19.12
3W86	**39**	1.50	0.145	0.184	1.00	19.13	15.13
3W87	**40**	1.43	0.155	0.187	0.67	15.53	10.62
3W88	**41**	1.40	0.140	0.166	0.67	21.44	11.03
4JD4	**42**	1.51	0.138	0.162	1.00	15.53	14.13
4JDB	**43**	1.82	0.169	0.211	1.00	26.24	20.87

B-factor: The average of B-factors.

### Interaction energy analysis

Interaction energy analysis was performed using the analytical tool Facio [39] based on pair interaction energy decomposition analysis, as proposed by Fedorov et al. [40]. This analysis using the FMO method provides a quantitative evaluation of hydrogen bonding and hydrophobic interactions that are important for ligand binding to a protein, as well as π-π, π–cation, and CH–π interactions, which require quantum chemical calculations [41]. These analyses are sometimes applied in structure-based drug design [42].

### Calculation procedure

All structures of the TcDHODH—compound complexes were visualized and computations were performed with hydrogenation. Optimization of the structures was performed only for the added hydrogen atoms, with all heavy atoms fixed at the positions given in the PDB using the CHARMM force field [43] by Discovery Studio (Accelrys, San Diego, CA). FMO calculation job files were generated using FMOutil version 2.1, and calculations were performed for each A-chain monomer using GAMESS [44] at the MP2/6-31G level. Alternate positions at except active site adopted a type-A conformation. Alternate positions in the active site adopted both. All calculations were performed with the TSUBAME2.5 supercomputer at Tokyo Tech (a HP Proliant SL390s G7 server with an Intel Xeon X5670 2.93-GHz processor). The approximate calculation time of each structure was 3 h at the MP2/6-31G level.

### Definition of pharmacophore

In this paper, we define a common pharmacophore as that having characteristics common to more than 80% conserved interactions between TcDHODH and compounds, and a particular pharmacophore as that having characteristics that have interactions stronger than −10 kcal mol^-1^ between TcDHODH and compounds.

## Results

### Analysis of interaction energies between TcDHODH and orotate and oxonate

**Fig 3:**
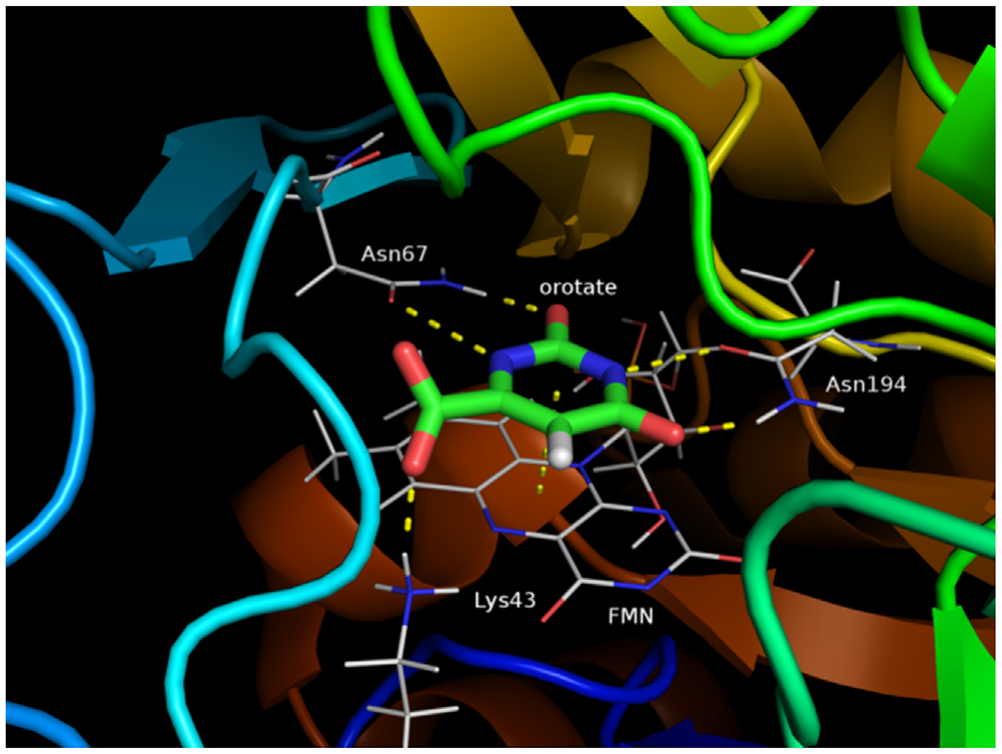
Orotate binding mode. Crystal structure of the TcDHODH—orotate complex active site. Hydrogen bond distance with the Lys43 amine group: 2.93 Å, with the Asn67 amine group: 3.05 Å, with the Asn67 carbonyl group: 2.82, with the Asn194 amine group: 3.32 and with the Asn194 carbonyl group: 2.92.

Orotate binds to the active center of TcDHODH, which is positioned parallel to an aromatic ring of FMN. Certain amino acid residues such as Lys43, Asn67, and Asn194 form hydrogen bonds with orotate and are important for substrate binding (Fig 3).

The side chain of Asn67 interacts with the 1-position hydrogen atom and 2-position carbonyl group of orotate, while the side chain of Asn194 side chain interacts with orotate’s 3-position hydrogen atom and 4-position carbonyl group through two hydrogen bonds. Moreover, the Lys43 side chain amide interacts with orotate’s 6-position carboxylate group.

**Fig 4:**
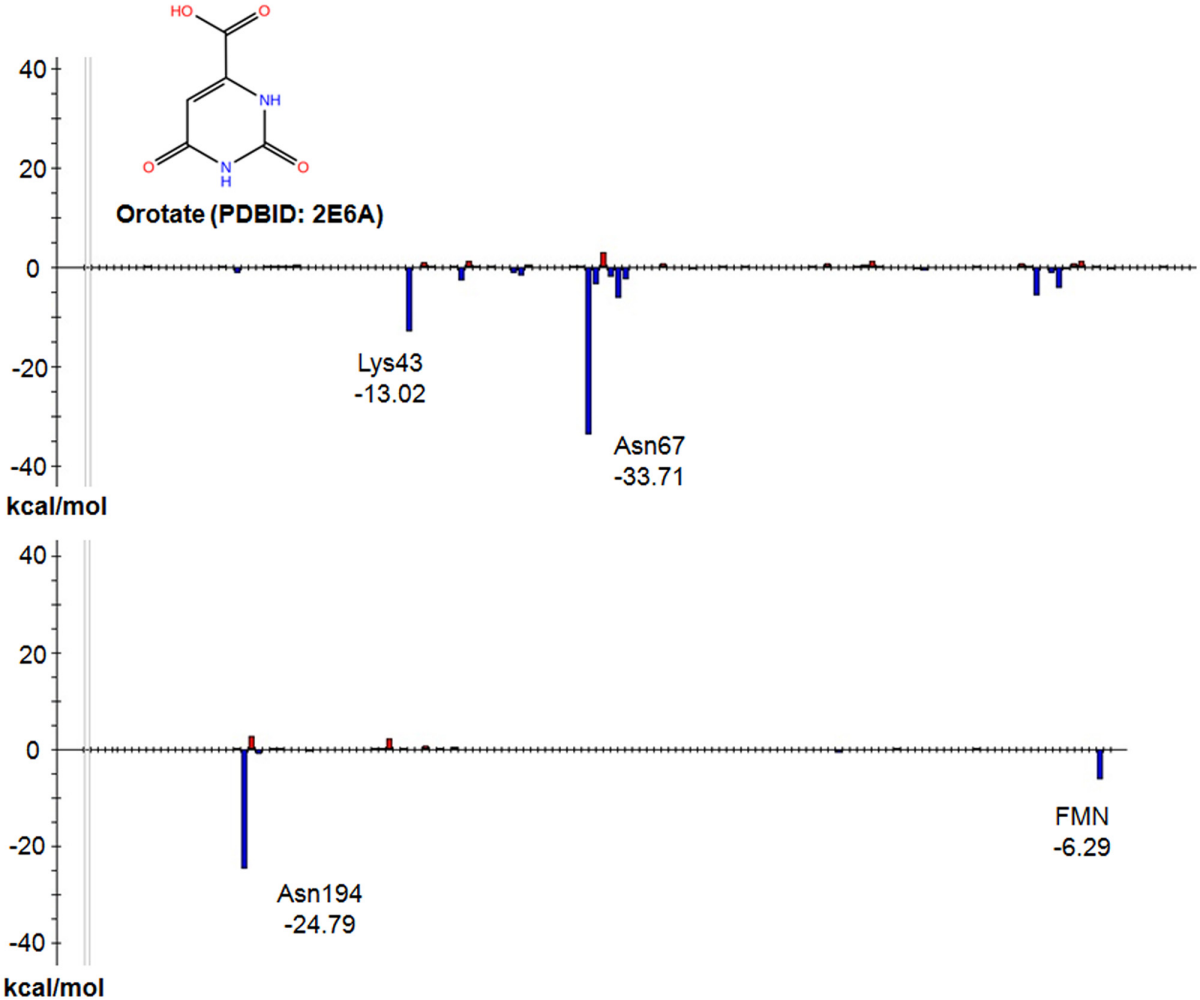
Interaction energy analysis of TcDHODH—orotate. The vertical axis shows the interaction energy between the ligand and each fragment, and the horizontal axis shows the fragment numbers. Fragments are numbered in order from N to C termini, followed by ligands, such as substrate and cofactor, to preserve the order described in the PDB file.

The FMO-based interaction energy analysis indicates that the hydrogen bonds with Lys43, Asn67 and Asn194 residues are important in this interaction (-13.02, -33.71, and -24.79 kcal mol^-1^, respectively), with interactions with Asn67 being strongest (Fig 4).

These findings indicate that Asn67 and Asn194 contribute to the specificity of orotate’s pyrimidine ring, and Lys43 contributes to the hydrogen bonding of orotate at the carboxylate moiety at the pyrimidine 6-position. The π-π interaction energy between orotate and FMN was −6.29 kcal mol^-1^ and contributed significantly to ligand binding (Fig 4). Given that Asn67 and Asn194 residues form two hydrogen bonds with orotate, large interaction energies would be predicted from this binding mode.

**Fig 5:**
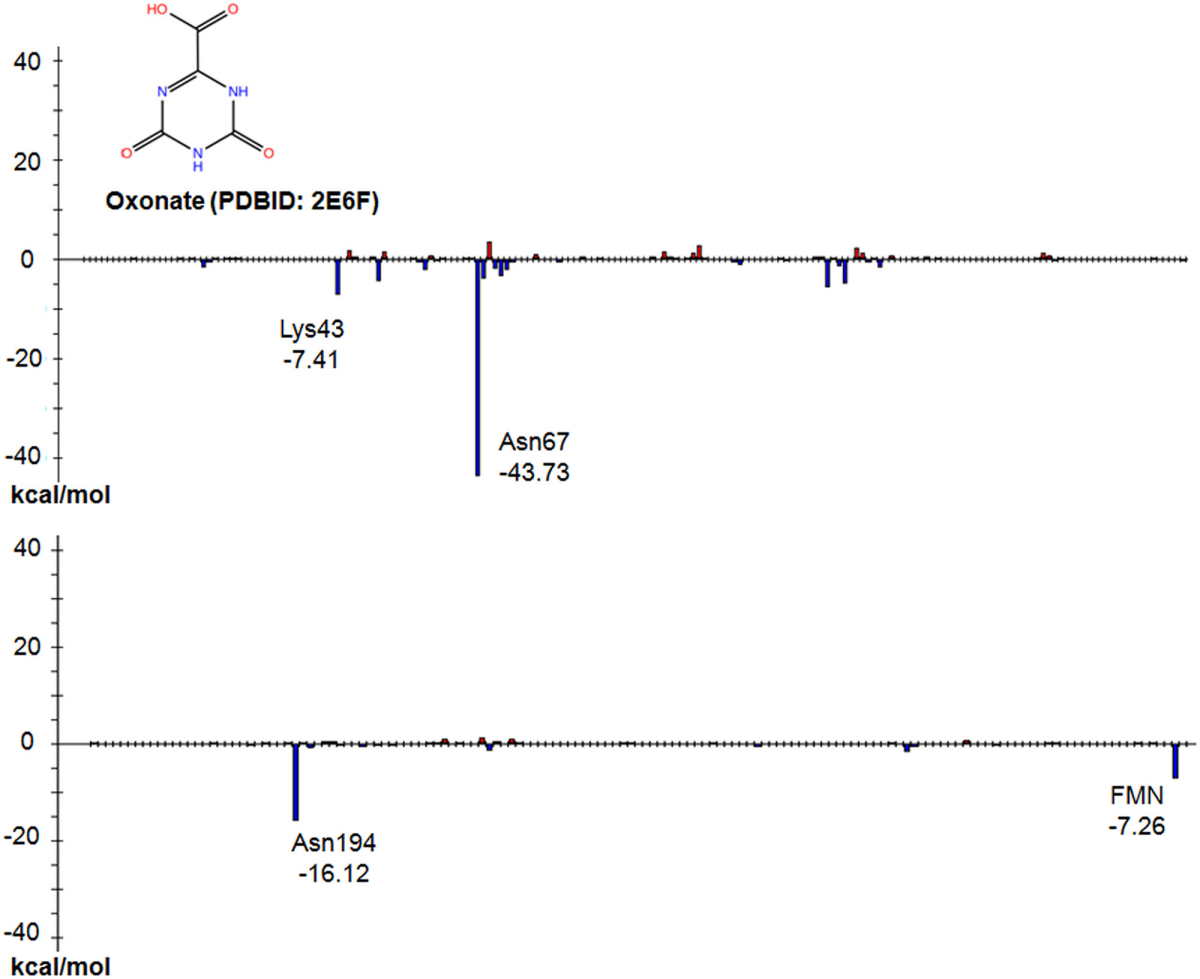
Interaction energy analysis of TcDHODH—oxonate. The vertical axis and horizontal axis are same as the Fig 4. (PDB ID: 2E6F, A-chain).

Oxonate, a well-known inhibitor of TcDHODH [15], also binds to the active center of TcDHODH, similarly to orotate. The FMO-based interaction energy analysis confirms the interaction with Lys43, Asn67, Asn194 and FMN as well as that of orotate (Fig 5: -7.41, -43.73, -16.21, and -7.26 kcal mol^-1^, respectively).

These results suggest that inhibitors targeting the active site of TcDHODH should preferentially have an aromatic moiety containing hydrogen bond donors and acceptors.

### Analysis of interaction energies between TcDHODH and orotate derivatives

**Fig 6:**
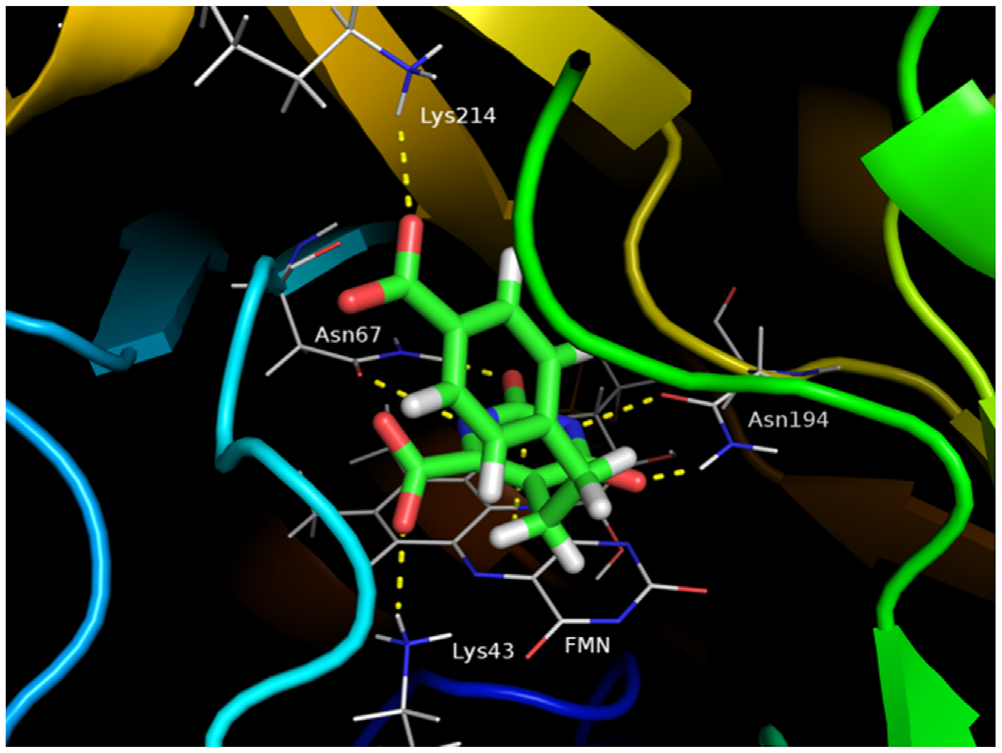
Binding mode of derivative 27. Crystal structures of the TcDHODH—derivative **27** complex active site. Hydrogen bond distance with the Lys43 amine group: 2.73 Å, with the Asn67 amine group: 2.89 Å, with the Asn67 carbonyl group: 2.96, with the Asn194 amine group: 2.86, with the Asn194 carbonyl group: 2.80, with the Lys214 residue: 2.91 Å.

Next, 43 orotate derivatives (Fig 2) predicted to form additional hydrogen bond and hydrophobic interactions were synthesized and their co-crystal structures were determined. These derivatives contain a functional group substituted at the pyrimidine 5-position through an ethylene or butylene linker. The binding mode between the active site of TcDHODH and derivative **27** is shown in Fig 6 as an example. Similarly to orotate, derivative **27** and other derivatives interact with Lys43, Asn67, Asn194 and FMN.

Furthermore, new interactions were predicted from the crystal structure, such as Lys214 by the introduction of a functional group at the pyrimidine 5-position.

These orotate derivatives were used to analyze the interaction energy by the FMO method. Table 2 shows the results of the interaction energy analyses for some amino acid residues and FMN. Lys43, Asn67, Asn194, and FMN showed strong interactions with the orotate moiety.

**Table 2:** Interaction energies between TcDHODH and orotate derivatives (kcal mol^-1^).

PDB ID	Derivative	Lys43	Asn67	Asn194	FMN	Lys214
3W1R	**1**	-11.40	-36.98	-25.47	-9.64	1.72
3W1T	**2**	-10.26	-35.07	-23.67	-8.39	3.17
3W1U	**3**	-11.01	-36.88	-30.16	-8.92	0.17
3W1X	**4**	-12.89	-38.06	-24.23	-9.78	2.66
3W2J	**5**	-7.78	-40.08	-25.61	-10.69	-11.89
3W2K	**6**	-9.97	-39.10	-26.14	-9.37	0.26
3W2L	**7**	-13.14	-35.55	-24.46	-8.65	4.41
3W2M	**8**	-8.87	-39.46	-26.45	-10.48	-7.13
3W2N	**9**	-13.00	-39.49	-27.22	-8.41	-2.00
3W2U	**10**	-5.28	-37.27	-24.90	-9.33	-7.48
3W3O	**11**	-14.69	-36.40	-28.38	-7.77	2.84
3W22	**12**	-12.59	-37.19	-27.77	-8.49	0.46
3W23	**13**	-8.46	-37.26	-24.82	-9.77	2.78
3W6Y	**14**	-14.43	-34.32	-25.60	-8.09	5.73
3W7C	**15**	-14.39	-36.21	-25.72	-8.73	3.13
3W7D	**16**	-15.93	-38.93	-28.17	-7.48	0.58
3W7E	**17**	-12.63	-39.04	-27.31	-8.50	1.59
3W7G	**18**	-2.77	-39.69	-23.95	-10.71	0.69
3W7H	**19**	-12.81	-38.30	-24.42	-8.36	1.51
3W7I	**20**	-16.99	-34.75	-23.45	-8.51	2.58
3W7J *	21*	-10.24	-39.80	-27.15	-9.62	1.88
3W7J **	21**	-9.52	-35.73	-26.05	-12.73	-16.50
3W7K	**22**	-17.90	-34.85	-29.04	-7.66	-3.67
3W7L	**23**	-5.54	-39.55	-24.21	-9.74	1.06
3W7M	**24**	-11.72	-39.73	-26.86	-7.00	4.99
3W7N	**25**	-13.58	-40.59	-26.87	-7.37	1.93
3W7O	**26**	-11.13	-37.11	-25.37	-9.26	2.05
3W7P	**27**	-8.50	-37.28	-25.71	-9.76	-13.55
3W7Q	**28**	-13.21	-32.89	-26.72	-7.85	-1.95
3W70	**29**	-17.93	-31.13	-27.90	-4.56	-0.07
3W71	**30**	-14.88	-37.71	-25.69	-8.27	5.48
3W72	**31**	-15.76	-38.29	-30.00	-8.21	0.78
3W73	**32**	-12.73	-39.97	-29.00	-8.74	-1.02
3W74	**33**	-10.35	-33.38	-27.55	-10.09	-3.62
3W75	**34**	-17.08	-40.20	-29.55	-8.96	3.53
3W76	**35**	-15.64	-39.26	-28.43	-9.97	4.56
3W83	**36**	-16.05	-25.20	-25.57	-6.43	0.84
3W84	**37**	-10.78	-34.59	-24.88	-7.99	4.40
3W85	**38**	-13.72	-34.61	-29.44	-8.75	-0.12
3W86	**39**	-11.87	-42.10	-29.49	-8.79	6.61
3W87	**40**	-15.37	-39.60	-27.92	-8.41	-3.40
3W88	**41**	-13.33	-39.38	-29.22	-9.34	-4.98
4JD4	**42**	-16.41	-38.66	-29.45	-8.39	-1.31
4JDB	**43**	-14.67	-36.77	-26.81	-8.33	1.24

* Lys214 alternative conformer A

** Lys214 alternative conformer B

The interaction energies between three amino acid residues and all orotate derivatives were strongly similar to those of orotate. Our analyses indicated that interactions between the orotate moiety and these three amino acid residues as well as FMN were strongly conserved for all of the derivatives. In contrast, some derivatives such as the alternative conformer B of **21** showed the strongest interaction with Lys214, followed by **27**, **5**, **10**, and **8** with interaction energies of -16.50, -13.55, -11.89, -7.48, and -7.13 kcal mol^-1^, respectively. The carboxylate group introduced in the phenyl moiety of **27** formed a hydrogen bond with the Lys214 residue (Fig 6). Derivatives **40** and **41** interacted with Lys214 with low energy despite the introduction of a carboxylate group on the aromatic ring moiety. Derivatives **40** and **41** were linked to a functional group at the pyrimidine 5-position through a butylene linker. In contrast, derivatives **21** and **27** interacted strongly (>10 kcal mol^-1^) when coupled with functional groups through an ethylene linker. These results suggest that the ethylene linker is appropriate for mediating interactions with Lys214.

### Pharmacophore obtained from calculation

From these results, intermolecular interactions with the Lys43, Asn67 and Asn194 by hydrogen bond and FMN through π-π interaction were obtained as a common pharmacophore in the orotate moiety and intermolecular interactions with the Lys214 by hydrogen bond obtained as particular pharmacophore at derivatives **5**, **21** and **27**.

## Discussion

This study provides a comprehensive FMO-based interaction analysis between TcDHODH residues and 43 orotate derivative inhibitors. Our analysis indicated that the orotate moiety formed hydrogen bonds with the Lys43, Asn67, and Asn194 residues of TcDHODH and a π-π interaction with FMN. These interactions were conserved in oxonate and orotate derivatives, and are also consistent with the magnitude of the interaction energy. From these results, the orotate moiety of orotate derivatives maintained these interactions whether or not functional groups were introduced at the pyrimidine ring 5-position. We accordingly expected a pharmacophore model containing molecules with hydrogen-bond donor/acceptor groups corresponding to Lys43, Asn67, and Asn194, such as amine, carbonyl, and carboxylate groups with an aromatic ring close to FMN.

**Fig 7:**
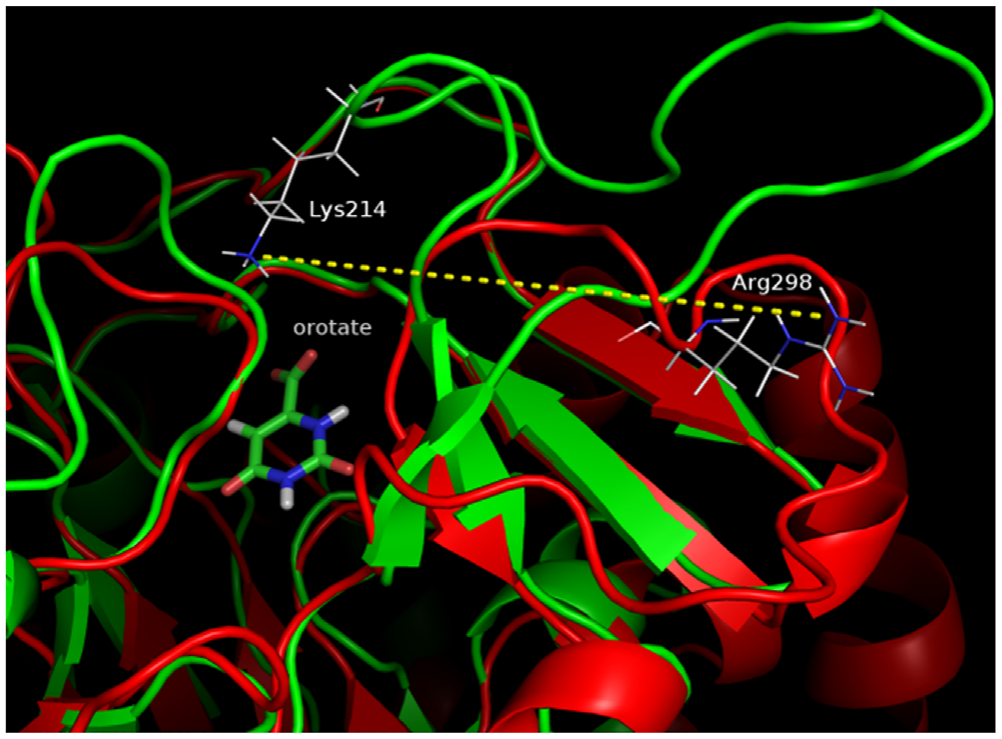
Comparison of loop structures. Alignment structure based on orotate (green ribbon: 2E6A, blue ribbon: 1D3G, distance between TcDHODH Lys214 and human DHODH Arg298: 19.5 Å). Superimposition was performed by “Align Binding Sites” in Maestro based on orotate.

In contrast, some derivatives containing an acceptor at a functional group form a hydrogen bond with the Lys214 residue. In particular, the energies of interaction between Lys214 and derivatives **5**, **21**, **27** were predicted to be stronger than -10 kcal mol^-1^. Furthermore, alignment analysis has shown that the TcDHODH Lys214 residue is replaced by Arg298 in human DHODH [15]. In their crystal structures, TcDHODH Lys214 and human DHODH Arg298 are each located in the loop connecting βF–βG and β6–βE [14–15]. These loop regions are poorly conserved within the family of DHODHs, an observation consistent with the difference in the loop structures (Fig 7); whereas, the amino group of TcDHODH Lys214 points towards the active site, the guanidium group of human DHODH Arg298 points to the opposite side, with a distance of 20 Å between the two.

Thus, even if TcDHODH Lys214 and human DHODH Arg298 are apparently conserved by alignment analysis, they are structurally not conserved. Consequently, orotate derivatives such as derivatives **5**, **21**, and **27** may specifically inhibit TcDHODH by interacting with the Lys214 residue. Thus, a hydrogen bond acceptor as a particular pharmacophore corresponding to Lys214 is necessary to inhibit TcDHODH selectively.

**Fig 8:**
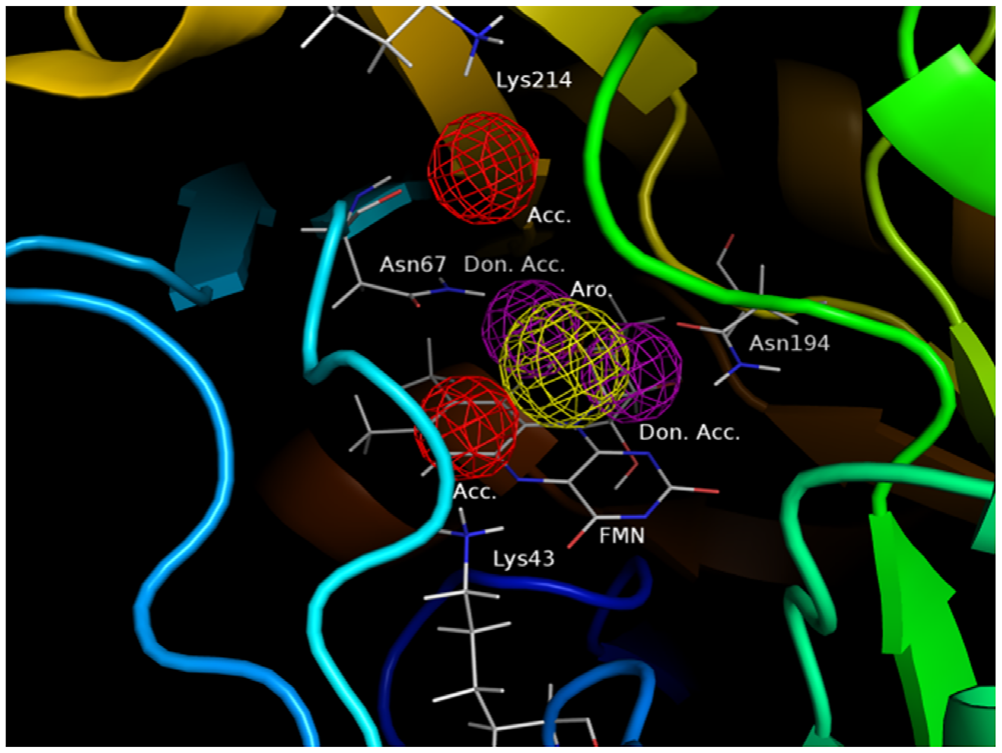
Pharmacophore inferred from interaction energy analysis. Acc.: Acceptor, Don.: Donor and Aro.: Aromatic.

In conclusion, we propose a pharmacophore model inferred from FMO-based interaction analysis. Hydrogen bond acceptor pharmacophores correspond to Lys43 and Lys214, hydrogen bond donor and acceptor pharmacophores correspond to Asn67 and Asn197, and an aromatic ring pharmacophore corresponds to FMN, indicating important characteristics of compounds expected to inhibit *Trypanosoma cruzi* DHODH (Fig 8).

These characteristics inferred from single-point FMO calculations for a large number of structures provide insights into the ligand—amino acid residue interactions important for pharmacophore modeling and may facilitate the development of TcDHODH—targeted anti-Chagas drugs.
